# Global child-friendly anti-TB medicines – where do we stand?

**DOI:** 10.5588/ijtldopen.25.0446

**Published:** 2025-09-10

**Authors:** H.S. Schaaf, D.T. Wademan, L.E. van der Laan

**Affiliations:** Desmond Tutu TB Centre, Department of Paediatrics and Child Health, Faculty of Medicine and Health Sciences, Stellenbosch University, Cape Town, South Africa.

**Keywords:** tuberculosis, dispersible, extemporaneous, formulation manipulation, pretomanid, delamanid, clofazimine, fluoroquinolones, bedaquiline

## Abstract

An increasing number of children are diagnosed and started on antituberculosis treatment. Despite progress in developing child-friendly antituberculosis formulations for drug-susceptible and drug-resistant TB, a single-medicine rifampicin dispersible tablet is still needed. Further, many child-friendly dispersible solid-tablet formulations are not available globally. Access challenges lead to formulation manipulation of adult tablets, including the development of extemporaneous solutions, supported by pharmacokinetic studies to dose young children. Preparing extemporaneous formulations need pharmacies and trained staff. Therefore, a need remains for global collaboration to prioritise child-friendly solid, dispersible, functionally scored TB formulations and to ensure equal access for all children with TB globally.

TB remains an important cause of morbidity and mortality in children and young adolescents, with an estimated 1.3 million children under 15 years of age developing TB globally in 2023, of which an estimated 25,000 to 32,000 children developed rifampicin-resistant (RR)-TB.^1,2^ Although children affected by TB are still under-diagnosed and under-reported, a growing number of children are diagnosed and started on treatment. Child-friendly anti-TB formulations for drug-susceptible (DS) and drug-resistant (DR)-TB are crucial. They ensure correct dosing for children, prevent possible underdosing from manipulation of adult formulations, reduce the risk of toxicity from inadvertent overdosing, and improve adherence to treatment through better acceptability for children and their caregivers, who are responsible for preparing and administering these medicines. Healthcare workers' hesitation to diagnose and treat paediatric DS- and DR-TB may be reduced by the availability of child-friendly, easily administered medicines.^3^ These formulations should be easy to prepare, administer, acceptable, palatable, achieve adequate absorption (bioavailability) and be accessible to children with TB.

Much progress has been made in producing child-friendly, mainly solid, water-dispersible TB medicines. Since 2016, paediatric first-line fixed-dose combination (FDC) dispersible tablets have been introduced in high-TB-burden countries and most medicines for DR-TB are currently available as dispersible tablets through the Stop TB Partnership Global Drug Facility (GDF) – see Table.^4–6^ Dispersible solid formulations are particularly well suited to low-income settings, as they are easier to store and transport than suspensions, which often require refrigeration. However, many middle- and high-income countries face two primary challenges to accessing child-friendly medicines: a limited number of child TB patients and costly registration processes preventing access through the GDF.^7–9^ Additionally, stringent national procurement policies – such as those in high-TB-burden settings like South Africa, which favour open tenders and direct manufacturer procurement – often stymie the efficient supply of these medicines. These access challenges lead to formulation manipulation of adult tablets to dose young children. Pharmacokinetic studies support the use of manipulated adult formulations (e.g., levofloxacin, moxifloxacin, clofazimine, bedaquiline, delamanid, rifapentine and isoniazid) for paediatric patients, showing favourable bioequivalence.^10–15^ Taneja et al.^16–19^ did further benchtop research to develop extemporaneous sugar and sugar-free suspensions from adult compounds, which are easy to prepare with compatible ingredients that are widely available and affordable in low- and middle-income countries (LMICs) and which enhance palatability. These suspensions of bedaquiline, delamanid, pretomanid (pretomanid not yet for use in children <14 years) and clofazimine were validated with mass spectrometry to ensure drug stability and retention at room temperature and 30^o^C over a 30-day period and stored in routinely available plastic medicine bottles. The four tested drugs in both the sugar and sugar-free suspensions all had good chemical and physical stability during 30-day study periods at room temperature and 30^0^C except clofazimine as a sugar-free suspension, which was only stable for 15 days. For both delamanid and bedaquiline the United States Pharmacopeia limits were not met for microbial counts at 30 days in the sugar-syrup formulations, therefore the liquid formulations of these two drugs can be extemporaneously prepared and stored for 15 days in simple syrup and 30 days in the sugar-free formulation.

**Table.** Anti-TB medicines: current adult/adolescent formulations and child-friendly formulations.

Medicine	Adult and adolescent formulations	Child-friendly formulations	Comments (gaps/challenges)
**First-line drugs: single medicines**			
Isoniazid (H)	100 mg; 300 mg Uncoated tablets	100mg dispersible tablets (DT)	Adult tablets may be manipulated
Rifampicin (R)	150 mg; 300 mg capsules	100mg DT (not yet developed) or 200mg/5ml suspensions	No dispersible tablets – a current need. Bioavailability of suspensions uncertain. Adult capsules cannot be manipulated
Pyrazinamide (Z)	400 mg; 500 mg Uncoated tablets	150mg DT	
Rifabutin (Rbt)	150 mg capsules	None	
Rifapentine (P)	150 mg; 300 mg Film coated tablets (FCT)	150mg DT	Used in TPT. Adult tablets may be manipulated
Ethambutol (E)	400 mg FCT	100mg DT	
**First-line drugs: fixed-dose combinations**			
RH	150/75 mg; 150/300 mg FCT	75/50 mg DT	
RHZ	Not available	75/50/150 mg DT	
RHZE	150/75/400/275 mg FCT	Not available	
HP	300/300 mg FCT	Not available	Used in TPT. Adult tablets may be manipulated
**Second-line drugs: medicines for drug-resistant TB**			
*Group A medicines* *			
Bedaquiline	100 mg Uncoated tablets	20 mg DT	Adult tablets may be manipulated
Levofloxacin	250 mg, 500 mg, 750 mg FTCs	100 mg DT	Adult tablets may be manipulated
Moxifloxacin	400 mg FCT	100 mg DT	Adult tablets may be manipulated
Linezolid	600 mg FCT	150 mg DT; 20mg/ml suspension	
*Group B medicines* *			
Clofazimine	50mg FCT or gel capsules; 100mg tablets or gel capsules	50 mg FCT or gel capsules	Tablets disperse well but not marketed as DT. Adult tablets may be manipulated
Cycloserine	250 mg capsules	125 mg capsules	
Terizidone	250 mg capsules		
*Group C medicines* *			
Delamanid	50 mg FCT	25 mg DT	Adult tablets may be manipulated
Ethionamide	250 mg FCT	125 mg DT	
Prothionamide	250 mg FCT		
Para-aminosalicylic acid (PAS)	4g granule/powder sachets or 1 g tablets	4g granule/powder	Measuring equal doses of granules and powder have different volumes
Meropenem	500 mg or 1 g vials, powder, for injection	500 mg vials, powder, for injections	To be used in combination with amoxicillin/clavulanate
Amikacin	500 mg/2 ml or 1 g /4ml vials for injection	100, 250, or 500 mg/2 ml vials for injection	Only use if no other options available – may cause irreversible hearing loss
**Other**			
Pretomanid	200 mg tablets	Not applicable	Not for children <14 years of age. Approved within regimens

* WHO rifampicin-resistant medicine groups.

DT = dispersible tablets; FCT = film coated tablet; TPT = TB preventive treatment.

These extemporaneous suspensions have practical advantages in that these suspensions are easy to prepare in a pharmacy, utilise adult formulations which are readily available, and can be stored without a fridge in routinely available plastic medicine bottles. However, several challenges remain for LMICs, such as the short shelf lives, the need for a pharmacy/dispensary under pharmacist supervision to prepare the extemporaneous suspensions and carrying bulky bottles of medicine which may leak or fall over. Additionally, the palatability and acceptability for these extemporaneous suspensions remain unknown. Taneja et al., also tested a simple device (XTEMP-R®) for converting tablets of adult formulation TB medicines into a homogeneous suspension for home use by caregivers.^20^ Qualitative research of the XTEMP-R® tool showed that, while it effectively addressed usability challenges related to drug preparation and administration, fundamental obstacles concerning medication palatability, nausea, and adverse-effects remain significant barriers. Additionally, this tool is not publicly available. Considering these challenges, solid, dispersible, functionally scored tablets remain the optimal formulation in LMICs.

In a palatability study evaluating moxifloxacin and linezolid in nearly 100 children aged 5 to 17 years, results indicated that manufacturers’ formulations do not always align with paediatric preferences.^21^ While no specific taste preference emerged among the linezolid options, children expressed a clear preference for a moxifloxacin flavour different from that planned by the manufacturers. This finding emphasises the importance of involving children in palatability assessments beyond in-house pharmaceutical testing prior to production, as such engagement may improve adherence and alleviate familial stress associated with medicine administration. A further challenge in child-friendly medicines is the formulation variability: trial-specific formulations evaluated for acceptability and bioavailability may not transition to routine care. Consequently, alternative manufacturers may secure the tender, leading to inconsistent acceptability (e.g. varying palatability) and/or bioavailability.

There is an urgent need for higher doses of rifampicin than those provided in the currently available dispersible child-friendly first-line FDC. Additionally, in child contacts of DS-TB or isoniazid mono-resistant TB, a 4-month regimen of rifampicin is an important option as preventive treatment. This could be addressed by the production of a functionally scored 100 mg dispersible rifampicin tablet, which is a WHO Paediatric Drug Optimization (PADO) priority. Current adult capsules (150 or 300 mg) are unsuitable for preparing suspensions, as their contents do not dissolve in water and have a bitter taste. Moreover, previous rifampicin suspensions failed to achieve target drug concentration exposures in children at the recommended dose.^22^ Higher doses of rifampicin may be critical in severe forms of TB disease such as TB meningitis and miliary TB.^23,24^

In LMICs TB medicines are often prepared and dispensed by healthcare workers at primary healthcare level, other than pharmacists or clinicians, who typically have less training or experience, especially with DR-TB treatment. Training to understand the responsibilities of timely ordering and stock management, as well as recognizing that medicines with the same generic names may differ due to variations in mg strength and palatability (e.g., adult tablet versus child-friendly formulations), should be conducted and regularly evaluated.^25^

In conclusion, there is an urgent need for global collaboration to prioritise child-friendly solid, dispersible, functionally scored TB formulations and to ensure equal access for all children with TB globally. Current data suggest manipulating adult formulations as a viable interim solution but addressing this global access barrier to child-friendly, solid, dispersible, functionally scored, medicines requires urgent action through further research, collaborative funding, streamlined registration and innovative manufacturing – see Box. In the meantime, this could possibly be achieved using manipulated adult formulations from the start, provided validated methods are developed to improve palatability and ensure equitable availability for all children.

Box. Research gaps and future needs regarding child-friendly anti-TB medicines.

Prioritise the development of child-friendly, solid, dispersible, functionally scored formulations for all new TB medicines, with paediatric palatability assessments conducted upfront.

Introduce mechanisms to incentivise pharmaceutical companies to develop paediatric TB compounds (e.g., financial and regulatory incentives, tax breaks, expedited approvals).

Establish pharmaceutical-philanthropic (not-for-profit organisation) partnerships to promote child-friendly TB medicine development.

Operational research to determine how well healthcare workers understand, manage and dispense child-friendly formulations to children with DS- and DR-TB

Operational research to determine the practicality of preparing child-friendly TB medicine suspensions in pharmacies/dispensaries or using novel home-based methods such as XTEMP-R®

Pragmatic studies comparing the acceptability and palatability of different child-friendly formulations (e.g., dispersible tablets to extemporaneous suspensions)

Psychosocial studies to understand how treatment administration in households affects uptake, acceptability and adherence.

Identify needs for scalable formulations, validated manipulation of adult drugs to improve acceptability and global access.

Streamlining procurement by limiting tender processes and procuring child-friendly TB medicines via the GDF.

Assess GDF procurement’s feasibility, cost-effectiveness, and adherence to WHO child-friendly formulations in LMICs, including policy barriers.
